# Calculations of actual corneal astigmatism using total corneal refractive power before and after myopic keratorefractive surgery

**DOI:** 10.1371/journal.pone.0175268

**Published:** 2017-04-12

**Authors:** Kyoung Yul Seo, Hun Yang, Wook Kyum Kim, Sang Min Nam

**Affiliations:** 1 Department of Ophthalmology, Institute of Vision Research, Eye and Ear Hospital, Severance Hospital, Yonsei University College of Medicine, Seoul, Korea; 2 SU Yonsei Eye Clinic, Seoul, Korea; 3 B&Viit bright eye center, Seoul, Korea; 4 Department of Ophthalmology, CHA Bundang Medical Center, CHA University, Seongnam, South Korea; Oklahoma State University Center for Health Sciences, UNITED STATES

## Abstract

**Purpose:**

To calculate actual corneal astigmatism using the total corneal refractive astigmatism for the 4-mm apex zone of the Pentacam (TCRP4astig) and keratometric astigmatism (Kastig) before and after photorefractive keratectomy or laser *in situ* keratomileusis

**Methods:**

Uncomplicated 56 eyes after more than 6 months from the surgery were recruited by chart review. Various corneal astigmatisms were measured using the Pentacam and autokeratometer before and after surgery. Three eyes were excluded and 53 eyes of 38 subjects with with-the-rule astigmatism (WTR) were finally included. The astigmatisms were investigated using polar value analysis. When TCRP4astig was set as an actual astigmatism, the efficacy of arithmetic or coefficient adjustment of Kastig was evaluated using bivariate analysis.

**Results:**

The difference between the simulated keratometer astigmatism of the Pentacam (SimKastig) and Kastig was strongly correlated with the difference between TCRP4astig and Kastig. TCRP4astig was different from Kastig in magnitude rather than meridian before and after surgery; the preoperative difference was due to the posterior cornea only; however, the postoperative difference was observed in both anterior and posterior parts. For arithmetic adjustment, 0.28 D and 0.27 D were subtracted from the preoperative and postoperative magnitudes of Kastig, respectively. For coefficient adjustment, the preoperative and postoperative magnitudes of Kastig were multiplied by 0.80 and 0.66, respectively. By arithmetic or coefficient adjustment, the difference between TCRP4astig and adjusted Kastig would be less than 0.75 D in magnitude for 95% of cases.

**Conclusions:**

Kastig was successfully adjusted to TCPR4astig before and after myopic keratorefractive surgery in cases of WTR. For use of TCRP4astig directly, SimKastig and Kastig should be matched.

## Introduction

Uncorrected refractive astigmatism, even as low as 1.00 D, can affect distance and near vision as well as patients’ quality of life.[1] The prevalence of refractive astigmatism increases in old age due to changes in the magnitude and axis of corneal astigmatism.[2–4] Therefore, accurate measurement of total corneal astigmatism in senile cataract patients before surgery is crucial to avoid significant astigmatism after removal of the crystalline lens. The common solution for correcting corneal astigmatism during cataract surgery is implantation of a toric intraocular lens (IOL) according to keratometric astigmatism (Kastig). Patients with regular corneal astigmatism ≥ 0.75 D may be considered for a toric IOL.[5] In traditional keratometry measuring only the anterior corneal surface, a fixed correlation between the anterior and posterior corneal surface is assumed and the standardized keratometric refractive index of 1.3375 is used.[3] However, the standardized keratometric refractive index is selected arbitrarily, and the net power of the cornea is less than the standardized keratometric power.[6] In addition, the relationship between the anterior and posterior corneal astigmatism is not fixed as a function of age:[3,7–9] the anterior corneal astigmatism is usually with-the-rule (WTR) astigmatism in younger age groups, but predominantly against-the-rule (ATR) astigmatism in older age groups. In contrast, the posterior corneal astigmatism remains relatively stable in magnitude and ATR axis, regardless of age.

Consequently, two types of adjustment to inaccurate Kastig have been suggested. The first is an arithmetic method, in which some diopters would be subtracted in WTR astigmatism and added for ATR astigmatism according to a nomogram.[10] The other is a coefficient method, in which Kastig is multiplied by the coefficient for each type of astigmatism.[11] However, both adjustment methods may not be perfect because the magnitude and axis of posterior corneal astigmatism are variable between individual patients.[12,13] To overcome the limitations of the adjustment methods, both anterior and posterior corneal astigmatisms are accurately measured in each patient, and the total corneal astigmatism is calculated. Postoperative refractive results after toric IOL implantation are improved using the total corneal astigmatism of the total corneal refractive power of the Pentacam.[13,14]

Calculation of toric IOL power in the modified cornea through keratorefractive surgery is more challenging. Keratorefractive surgeries for myopia correction make changes only on the anterior surface of the cornea; as such, the posterior power in the keratometric power is more overestimated; additionally, the relationship between the anterior and posterior corneal astigmatism may be more variable.[15,16] To our knowledge, toric IOL calculation in the modified cornea has not been investigated in detail, and no adjustment method has been reported to date. The total corneal refractive power of 4-mm apex zone by Pentacam (TCRP4) is a possible solution, because it can accurately measure the surgically induced changes in manifest refraction after corneal laser surgery.[17] In contrast to the keratometric power, TCRP4 does not rely on any prior assumptions about the corneal shape but is the most realistic means of determining the corneal power.[17] In addition, the TCRP4 method, combining the equivalent K reading derived from TCPR4 and the Holladay 2 formula, shows good predictive capability of the IOL power calculation for the modified cornea after myopic keratorefractive surgery.[18]

In this study, the total corneal refractive power astigmatism for the 4-mm apex zone (TCRP4astig) was compared with Kastig and the causes of the difference between them were investigated before and after corneal laser surgery for myopia. The efficacies of arithmetic and coefficient methods were compared in adjustment of Kastig to TCRP4astig, preoperatively and postoperatively. This study confirmed previous reports on the actual corneal astigmatism calculation in the unmodified cornea and provided insights for the modified cornea.

## Materials and methods

### Subjects

Myopic patients undergoing photorefractive keratectomy (PRK) or laser *in situ* keratomileusis (LASIK) at B&VIIT Eye Center, Seoul, Korea, between 2009 and 2011, were randomly selected and reviewed. Surgery was performed using the Allegretto Wave Eye-Q laser (Wavelight Laser Technologie AG) or the Amaris excimer laser (Schwind Eye-Tech-Solutions GmbH and Co. KG). The best corrected visual acuity, the corneal power of the autokeratometer (ARK-530A; Nidek, Gamagori, Japan), and Pentacam measurements (Pentacam or Pentacam HR; Oculus, Wetzlar, Germany) were obtained preoperatively and postoperatively.

Eyes fulfilling the following criteria were included: postoperative measurements after more than 6 months from surgery; corrected visual acuity not worse than 20/20 preoperatively and postoperatively; healthy cornea before surgery and not accompanied by any postoperative complications; absolute difference between cornea front power of the Pentacam (simulated keratometer reading, SimK) and the corresponding keratometric power of the autokeratometer no more than 0.5 D preoperatively and postoperatively. The exclusion criteria were as follows: absolute difference in astigmatic magnitude between the simulated keratometer astigmatism (SimKastig) and Kastig more than 0.5 D preoperatively or postoperatively; absolute difference in astigmatic steep meridian between SimKastig and Kastig more than 10° preoperatively or postoperatively. This retrospective study was approved by the Institutional Review Board, CHA University, and adhered to the tenets of the Declaration of Helsinki.

### Cornea astigmatism measurement

The Pentacam was operated in 25-image mode, in which the rotating camera acquires 25 scans within 2 s. Every Pentacam in this study was calibrated and technically supported by the manufacturer. Several types of corneal astigmatism were detected, as described below and in Table 1.

**Table 1:** Abbreviations and their definitions of various corneal powers and astigmatisms.

Abbreviation	Full term	Instrument	Definition
Kastig	Keratometric astigmatism	Autokeratometer	Corneal astigmatism related to keratometer reading; n = 1.3375
KBastig	Keratometric back astigmatism	Autokeratometer	Estimated back corneal astigmatism using keratometer reading, the standardized keratometric index (1.3375) and the refractive index of the cornea (1.376)
SimK	Simulated keratometer reading	Pentacam	Front simulated keratometer reading on a ring in 15° around the anterior corneal apex; n = 1.3375
SimKastig	Simulated keratometer astigmatism	Pentacam	Corneal astigmatism related to SimK
Bastig	Simulated keratometer back astigmatism	Pentacam	Back corneal astigmatism related back simulated keratometer reading on a ring in 15° around the corneal apex; n = 1.336–1.376
TCRP4	Total corneal refractive power for the 4-mm diameter zone with the apex center	Pentacam	Realistic corneal power calculated by ray tracing according to Snell’s law through the anterior and posterior corneal surfaces; the value for a 4-mm diameter zone with the apex center is chosen; n = 1 for air, 1.376 for the cornea, 1.336 for aqueous humor
TCRP4astig	Total corneal refractive astigmatism for the 4-mm diameter zone with the apex center	Pentacam	Realistic corneal astigmatism related to TCRP4
RP4astig	Front refractive astigmatism for the 4-mm diameter zone with the apex center	Pentacam	Corneal astigmatism related to front refractive power which is calculated by ray tracing according to Snell’s law through the anterior corneal surface; the value for a 4-mm diameter zone with the apex center is chosen; n = 1.3375

n = refractive index

For TCRP4astig, [17] [7] zone, apex, and total corneal refractive power options were selected in the power distribution display of the Pentacam software (version 1.18r04), and the zone diameter was set to 4.0 mm. The magnitude of TCRP4astig was calculated by K2 − K1 diopter in “power calculation in actual zone” and the meridian of K2 was read as the meridian of TCRP4astig.

For RP4astig,[19] front corneal refractive power, zone, apex, and 4.0-mm zone diameter options were selected in the power distribution display of the Pentacam software; the magnitude and meridian of RP4astig were determined in the same manner as for TCPR4astig.

KBastig was calculated from Kastig using using the Gaussian optics formula.[20]

Back power

$$\begin{array}{l} =\text{Keratometric power }\left(\text{K}\right)-\text{Front power }+\frac{d}{n}\;\left(\text{Front power}\;\times \;\text{Back power}\right)\\ \approx \text{K}-\text{Front power}\\ =\text{K}-\;\left(1.376-1\right)/((1.3375-1)/K)\\ =\text{K }(1-\left(1.376-1\right)/\left(1.3375-1\right)) \end{array}$$

(d: corneal thickness (m), n: corneal refractive index; (d/n) × (Front power × Back power) is as small as −0.13 D in normal eyes with 7.5-mm anterior corneal radius)[21])

$$\begin{array}{l} \text{KBastig}=\text{Bs}-\text{Bf }\\ =\left(\text{Ks}-\text{Kf}\right)\left(1-\left(1.376-1\right)/\left(1.3375-1\right)\right)\\ =\text{Kastig}\times \left(1-\left(1.376-1\right)/\left(1.3375-1\right)\right) \end{array}$$ (1)

(Bs: back power in the steep meridian, Bf: back power in the flat meridian, Ks: keratometric power in the steep meridian, Kf: keratometric power in the flat meridian)

### Assessment of corneal astigmatism

Net astigmatism is given as (M@α°), where *M* is the magnitude in diopters and *α* is the meridian in degrees. For any mathematical conversion such as subtraction or averaging of astigmatisms, a net astigmatism is transformed into two orthonormal polar values, the curvital and torsional powers, with units in diopters separated by an arch of 45°, KP(Φ) and KP(Φ + 45), respectively.[22–24]

$$\begin{array}{l} \text{KP}\left(\Phi \right)=\text{ curvital power }=\text{M cos}\left(2\left(\alpha -\Phi \right)\right)\\ \text{KP}\left(\Phi +45\right)=\text{ torsional power}=\text{M sin}\left(2\left(\alpha -\Phi \right)\right) \end{array}$$

The curvital power, KP(Φ), is the power acting along the reference meridian Φ, and the torsional power, KP(Φ + 45), is the power twisting the astigmatic direction toward either meridian, (Φ + 45) or (Φ—45). Calculations are performed with polar values; the results may be reconverted to net astigmatism by the known equations.

$$\text{M}=\;\sqrt{\text{KP}{\left(\Phi \right)}^{\text{2}}{+\text{KP}(\Phi +45)}^{\text{2}}\;}$$

$$\alpha \;=\text{ arctan}\left(\frac{\text{M}-\text{KP}\left(\Phi \right)}{\text{KP}\left(\Phi +45\right)}\right)\Phi +\text{p}\cdot 180$$

P is an integer and determines the periodic function for choosing an astigmatic meridian between zero and 180 degrees.

### Statistical analysis

Using polar values, univariate analysis was performed with calculation of univariate means and the *t* test. Bivariate analysis of polar values was performed with Hotelling’s T^2^ multivariate *t* test.[22]

$$T^{2}=\left(\frac{1}{1-r^{2}}\right)\left(t_{1}^{2}-2rt_{1}t_{2}+t_{2}^{2}\right)$$

where t_1_ and t_2_ are the paired t-values for univariate analysis of KP(Φ) and KP(Φ + 45). r is a correlation coefficient between ΔKP(Φ) and ΔKP(Φ + 45). The test statistics is transformed to an F-test with (2, n − 2) degrees of freedom as follow:

$$F\left(2,n-2\right)=\;\frac{n-2}{n-1}\frac{T^{2}}{2}$$

In addition, the bivariate 95% normal region for observations was graphically reported.[22] The bivariate normal distribution, reported in the (y1, y2) coordinate system, appears as:

$${\left(\frac{y1}{s_{y1}}\right)}^{2}+{\left(\frac{y2}{s_{y2}}\right)}^{2}=\chi^{2}-\left(P\right),\;dof=2$$

For a 95% confidence limit the χ^2^ (chi-square) value with two degrees of freedom (dof) is 5.991. If 0 < |r| < 1, y1, y2, s_y1_, s_y2_ are calculated as follow:

$$\epsilon =\;\frac{1}{2}arctan\frac{2rs_{KP\left(\Phi \right)}s_{KP\left(\Phi +45\right)}}{s_{KP\left(\Phi \right)}^{2}-s_{KP\left(\Phi +45\right)}^{2}}+p\cdot 90$$

where r is a correlation coefficient between KP(Φ) and KP(Φ + 45); s_KP(Φ)_ is standard deviation for KP(Φ);s_KP(Φ + 45)_ is standard deviation for KP(Φ + 45); p is an integer for selecting ϵ in the interval from 0–90°.

$$y1=\left(KP\left(\Phi \right)-\bar{KP\left(\Phi \right)}\right)\text{cos }\epsilon +\left(\text{KP}\left(\Phi +45\right)-\bar{KP\left(\Phi +45\right)}\right)\text{sin }\epsilon$$

$$y2=-\left(KP\left(\Phi \right)-\bar{KP\left(\Phi \right)}\right)\text{sin }\epsilon +\left(\text{KP}\left(\Phi +45\right)-\bar{KP\left(\Phi +45\right)}\right)\text{cos }\epsilon$$

where $\bar{\text{KP}\left(\Phi \right)}$ is mean of KP(Φ); KP(Φ+45) is mean of KP(Φ + 45).

$${\left(s_{y1}+s_{y2}\right)}^{2}=s_{KP\left(\Phi \right)}^{2}+s_{KP\left(\Phi +45\right)}^{2}+2rs_{KP\left(\Phi \right)}s_{KP\left(\Phi +45\right)}\sqrt{1-r^{2}}$$

$${\left(s_{y1}-s_{y2}\right)}^{2}=s_{KP\left(\Phi \right)}^{2}+s_{KP\left(\Phi +45\right)}^{2}-2rs_{KP\left(\Phi \right)}s_{KP\left(\Phi +45\right)}\sqrt{1-r^{2}}$$

To solve for s_y1_ and s_y2_, their signs are determined by the following relations:

$$\text{For r}>0,\left({\text{s}}_{\text{y1}}-{\text{ s}}_{\text{y2}}\right)>0;\text{ for r}<0,\left({\text{s}}_{\text{y1}}-{\text{ s}}_{\text{y2}}\right)<0$$

F-test for the total standard deviation was conducted.[22] The total variation (${\text{s}}_{\text{total}}^{\text{2}}$) is the sum of the variances for the two polar values.

$$s_{total}^{2}=s_{KP\left(\Phi \right)}^{2}+s_{KP\left(\Phi +45\right)}^{2}$$

Microsoft Excel for Mac 2011 (version 14.5.2; Microsoft, Inc.), IBM SPSS Statistics (version 22; International Business Machines Corp.), MedCalc (version 12.7.7.0; MedCalc Software), SigmaPlot (version 12.0; Systat Software, Inc.), Grapher (version 2.5, Apple Inc.), and GraphPad Prism (version 6.01, GraphPad Software; Inc.) were used for statistical calculations and graphical analyses.

## Results

Three eyes (one preoperative eye, two postoperative eyes) were excluded, because the steep meridians of Kastig were outside the range of 60°–120°. Finally, 53 eyes (29 right and 24 left) of 38 subjects (9 men and 29 women) with WTR astigmatism (steep meridian in 60°—120°) preoperatively and postoperatively were included in the study. The mean age of subjects was 26.2 ± 4.3 (SD) years. The mean sphere equivalent of manifest refraction was −4.92 ± 1.80 (SD) D. Twenty-five eyes underwent PRK and the other 28 eyes underwent LASIK. The geometric means of planned optical zone diameter for PRK and LASIK were 6.5 mm (6.4–6.5 mm, 95% confidence interval, CI; minimum 6.3, maximum 6.8 mm) and 6.6 mm (6.5–6.6 mm, 95% CI; minimum 6.4, maximum 6.7 mm), respectively. Postoperative measurements were performed after a median of 312 days (minimum 190, maximum 813 days).

After surgery, the anterior surface-based astigmatisms (Kastig, SimKastig, and RP4astig) and TCRP4astig were changed, but the back astigmatism (Bastig) remained the same as the preoperative value (Table 2). However, KBastig was lower after surgery because it was calculated from Kastig (Table 2).

**Table 2:** Various mean net astigmatisms of preoperative and postoperative cornea. Bivariate significance tests for differences between preoperative and postoperative astigmatisms of each subject were performed on 0 diopter @ 0° using Hotelling’s T^2^.

	Keratometer		Pentacam			
	Kastig	KBastig	SimKastig	RP4astig	Bastig	TCRP4astig
Preoperative cornea (diopter @ degree)	1.47 @ 88	0.17 @ 178	1.4 @ 89.5	1.5 @ 90	0.4 @ 0.8	1.2 @ 89
Postoperative cornea (diopter @ degree)	0.83 @ 88	0.09 @ 178	0.9 @ 86.8	0.9 @ 87	0.5 @ 179.7	0.5 @ 86
*P*-value	< 0.001	< 0.001	< 0.001	< 0.001	0.301	< 0.001

Kastig = keratometric astigmatism; KBastig = keratometric back astigmatism; SimKastig = simulated keratometer astigmatism; RP4astig = front refractive astigmatism in the 4-mm diameter zone with the apex center; Bastig = simulated keratometer back astigmatism; TCRP4astig = total corneal refractive astigmatism in the 4-mm diameter zone with the apex center.

When the Kastig meridian (Φ) was set as the reference meridian, both KP(Φ) (curvital power) and KP(Φ + 45) (torsional power) of SimKastig − Kastig were strongly positively correlated with those of (RP4astig − Kastig) and (TCRP4astig − Kastig) before and after surgery (Table 3). Therefore, agreement between SimKastig and Kastig affected the difference between Kastig and other Pentacam astigmatisms, such as RP4astig and TCPR4astig. In this study, SimKastig was not significantly different from Kastig both preoperatively and postoperatively (Table 4).

**Table 3:** Univariate analysis of preoperative and postoperative Pearson’s r with SimKastig − Kastig.

	For Curvital Power, KP(Φ) (95% confidence interval)		For Torsional Power, KP(Φ + 45) (95% confidence interval)	
RP4astig − Kastig				
Preoperative	0.9541 (0.9215–0.9734)	*P* < 0.001	0.9802 (0.9658–0.9886)	*P* < 0.001
Postoperative	0.9525 (0.9187–0.9724)	*P* < 0.001	0.8643 (0.7751–0.9197)	*P* < 0.001
TCRP4astig − Kastig				
Preoperative	0.9009 (0.8336–0.9418)	*P* < 0.001	0.9256 (0.8739–0.9566)	*P* < 0.001
Postoperative	0.8172 (0.7019–8907)	*P* < 0.001	0.7130 (0.5484–0.8244)	*P* < 0.001

SimKastig = simulated keratometer astigmatism; Kastig = keratometric astigmatism; RP4astig = front refractive astigmatism in the 4-mm diameter zone with the apex center; TCRP4astig = total corneal refractive astigmatism in the 4-mm diameter zone with the apex center; Φ = reference plane, steep meridian of Kastig in degrees.

**Table 4:** Mean net astigmatisms of difference between various astigmatisms of the Pentacam and the Keratometer before and after surgery. Bivariate significance test on 0 diopter @ (Φ + 0)° was performed using Hotelling’s T^2^.

	Preoperative cornea (diopter @ degree)		Postoperative cornea (diopter @ degree)	
SimKastig − Kastig	0.08 @ (Φ + 67)	*P* = 0.076	0.04 @ (Φ +169)	*P* = 0.428
RP4astig − Kastig	0.07 @ (Φ + 47)	*P* = 0.244	0.09 @ (Φ + 2)	*P* = 0.045
TCRP4astig − Kastig	0.28 @ (Φ + 87)	*P* < 0.001	0.28 @ (Φ + 93)	*P* < 0.001
Bastig − KBastig	0.27 @ (Φ + 95)	*P* < 0.001	0.34 @ (Φ + 93)	*P* < 0.001

SimKastig = simulated keratometer astigmatism; Kastig = keratometric astigmatism; RP4astig = front refractive astigmatism in the 4-mm diameter zone with the apex center; TCRP4astig = total corneal refractive astigmatism in the 4-mm diameter zone with the apex center; Bastig = simulated keratometer back astigmatism; KBastig = keratometric back astigmatism; Φ = reference plane, steep meridian of Kastig in degrees.

### Reason for the difference between TCRP4astig and Kastig

The difference between TCRP4astig and Kastig was related to KP(Φ) rather than KP(Φ + 45) before and after surgery (Table 5). Preoperatively, RP4astig as an anterior element of TCRP4astig was not significantly different from Kastig, but Bastig as a posterior element of TCRP4astig was 0.3 D less in KP(Φ) than KBastig (Tables 4 and 5). Although Bastig differed in KP(Φ + 45) from KBastig, this effect failed to influence the difference between TCRP4astig and Kastig (Table 5).

**Table 5:** Univariate analysis of the mean preoperative and postoperative values of TCRP4astig − Kastig, Bastig − KBastig, and RP4astig − Kastig.

	Curvital Power (D), KP(Φ) ± standard deviation	*P*-value^a^	Torsional Power (D), KP(Φ + 45) ± standard deviation	*P*-value^a^
TCRP4astig − Kastig				
Preoperative	−0.28 ± 0.26	< 0.001	0.03 ± 0.27	0.382
Postoperative	−0.27 ± 0.27	< 0.001	−0.02 ± 0.21	0.403
*P*-value^b^	0.977		0.270	
Bastig − KBastig				
Preoperative	−0.27 ± 0.12	< 0.001	−0.04 ± 0.13	0.016
Postoperative	−0.34 ± 0.15	< 0.001	−0.03 ± 0.16	0.172
*P*-value^b^	< 0.001		0.520	
RP4astig − Kastig				
Preoperative	0.00 ± 0.22	0.901	0.07 ± 0.28	0.092
Postoperative	0.09 ± 0.26	0.013	0.01 ± 0.17	0.791
*P*-value^b^	0.049		0.129	

TCRP4astig = total corneal refractive astigmatism in the 4-mm diameter zone with the apex center; Kastig = keratometric astigmatism; Bastig = simulated keratometer back astigmatism; KBastig = keratometric back astigmatism; RP4astig = front refractive astigmatism in the 4-mm diameter zone with the apex center; Φ = reference plane, steep meridian of Kastig in degrees.

^a^compared to zero (*t* test).

^b^compared between preoperative and postoperative values (paired *t* test).

**Fig 1:**
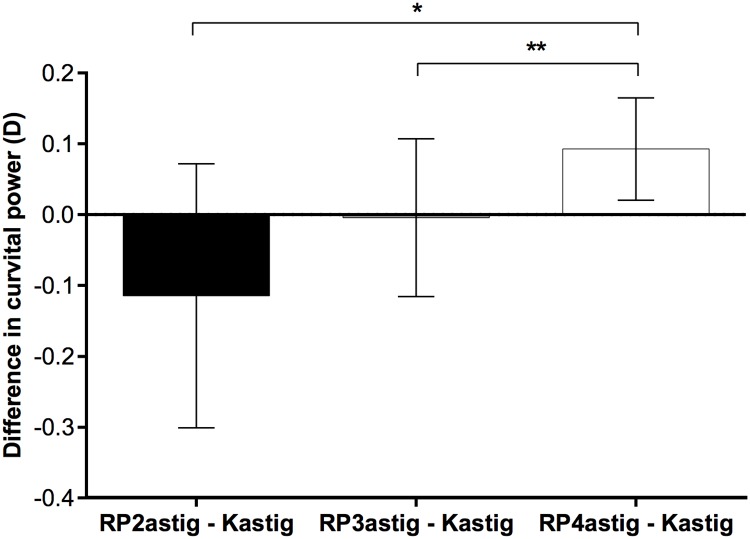
Postoperative comparison of differences in curvital power between front refractive astigmatism and keratometric astigmatism (Kastig). The differences were calculated for 2-, 3-, and 4-mm zone front refractive astigmatisms (RP2astig − Kastig, RP3astig − Kastig, RP4astig − Kastig, respectively). The curvital power was calculated along the meridian of Kastig. The differences were significant, especially for RP4astig − Kastig (*P* = 0.005, repeated ANOVA; *P = 0.014, **P = 0.003, Turkey’s multiple comparisons test). Error bars represent the 95% confidence interval of the mean.

Postoperatively, Kastig became less in KP(Φ) than RP4astig, but was statistically the same in KP(Φ + 45) as RP4astig (Table 5). No difference was observed in KP(Φ) between the front refractive astigmatism and Kastig for 2- and 3-mm zone refractive power (Fig 1). Bastig was 0.3 D less in KP(Φ) than KBastig, but not significantly different in KP(Φ + 45) from KBastig (Table 5).

**Fig 2:**
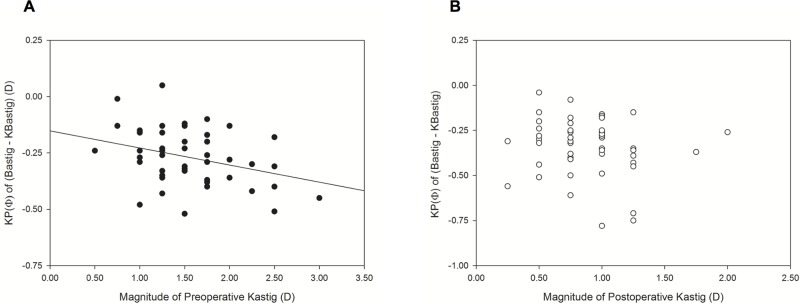
Scatter plots and a regression line between the magnitude of keratometric astigmatism (Kastig) and the curvital power of (Bastig—KBastig) along Φ before (A) and after (B) surgery. Bastig, simulated keratometer back astigmatism of the Pentacam; KBastig, keratometric back astigmatism; Φ, steep meridian of Kastig.

Therefore, KP(Φ) of (Bastig—KBastig) was the common reason for the difference between TCRP4astig and Kastig before and after surgery. Preoperative KP(Φ) of (Bastig—KBastig) was negatively correlated with Kastig_magnitude_ (*P* = 0.017, Pearson’s r = − 0.3280) (Fig 2A). In contrast, postoperative KP(Φ) of (Bastig—KBastig) did not show correlation with Kastig_magnitude_ (*P* = 0.464, Spearman’s rank correlation) (Fig 2B).

### Adjustments to Kastig to fit TCRP4astig

**Fig 3:**
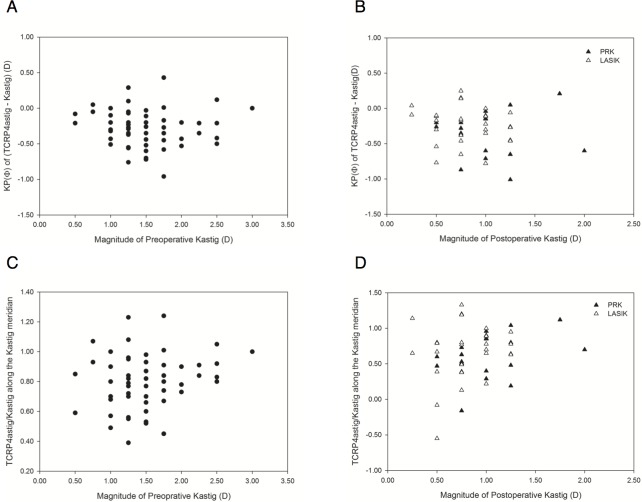
Scatter plots between the magnitude of keratometric astigmatism (Kastig) and adjustment factors before (A and C) and after (B and D) surgery. TCRP4astig, 4-mm-apex zone total corneal refractive astigmatism; Φ, steep meridian of Kastig; KP(Φ), curvital power along Φ meridian; PRK, photorefractive keratectomy, LASIK, laser in situ keratomileusis

As Kastig and TCRP4astig were different in KP(Φ), but not in KP(Φ + 45), the KP(Φ) of Kastig (magnitude of Kastig, Kastig_magnitude_) was targeted for adjustment to fit TCRP4astig (Table 5). Two adjustment types were evaluated. First, arithmetic adjustment added the mean KP(Φ)_TCRP4astig–Kastig_ to Kastig_magnitude_ (KP(Φ)_TCRP4astig–Kastig_ = KP(Φ) of (TCRP4astig − Kastig)) (Tables 5 and 6). Second, coefficient adjustment multiplied Kastig_magnitude_ by the mean (KP(Φ)_TCRP4astig_/Kastig_magnitude_) (KP(Φ)_TCRP4astig_ = KP(Φ) of TCRP4astig) (Table 6). Neither KP(Φ)_TCRP4astig–Kastig_ nor KP(Φ)_TCRP4astig_/Kastig_magnitude_ was correlated with Kastig_magnitude_ preoperatively (*P* = 0.657, *P* = 0.156, respectively; Pearson’s correlation) or postoperatively (*P* = 0.477, *P* = 0.113, respectively; Spearman’s rank correlation) (Fig 3). Postoperative KP(Φ)_TCRP4astig–Kastig_ and KP(Φ)_TCRP4astig_/Kastig_magnitude_ were independent of optical zone diameters (*P* = 0.698, Pearson’s correlation; *P* = 0.918, Spearman’s rank correlation, respectively).

**Table 6:** Comparison between arithmetic and coefficient adjustment methods before and after keratorefractive surgery.

	Arithmetic adjustment	Coefficient adjustment
Preoperative eyes (Kastig_magnitude_ range: 0.50–3.00 D)		
Adjustment factor (95% confidence interval)	− 0.28 (− 0.35 to − 0.20)	0.80 (0.75–0.86)
Calculations of adjusted Kastig	(Kastig_magnitude_ − 0.28 D)@Φ	(Kastig_magnitude_ × 0.80)@ Φ
Postoperative eyes (Kastig_magnitude_ range: 0.25–2.00 D)		
Adjustment factor (95% confidence interval)	− 0.27 (− 0.35 to − 0.20)	0.66 (0.56–0.75)
PRK	− 0.30 (− 0.42 to– 0.17)	0.66 (0.53 to 0.79)
LASIK	− 0.25 (− 0.35 to– 0.16)	0.65 (0.50 to 0.80)
Calculations of adjusted Kastig	(Kastig_magnitude_ − 0.27 D) @Φ	(Kastig_magnitude_ × 0.66) @Φ

Kastig = keratometric astigmatism; Kastig_magnitude_ = magnitude of Kastig; Φ = steep meridian of Kastig in degrees; PRK, photorefractive keratectomy, LASIK, laser in situ keratomileusis

**Fig 4:**
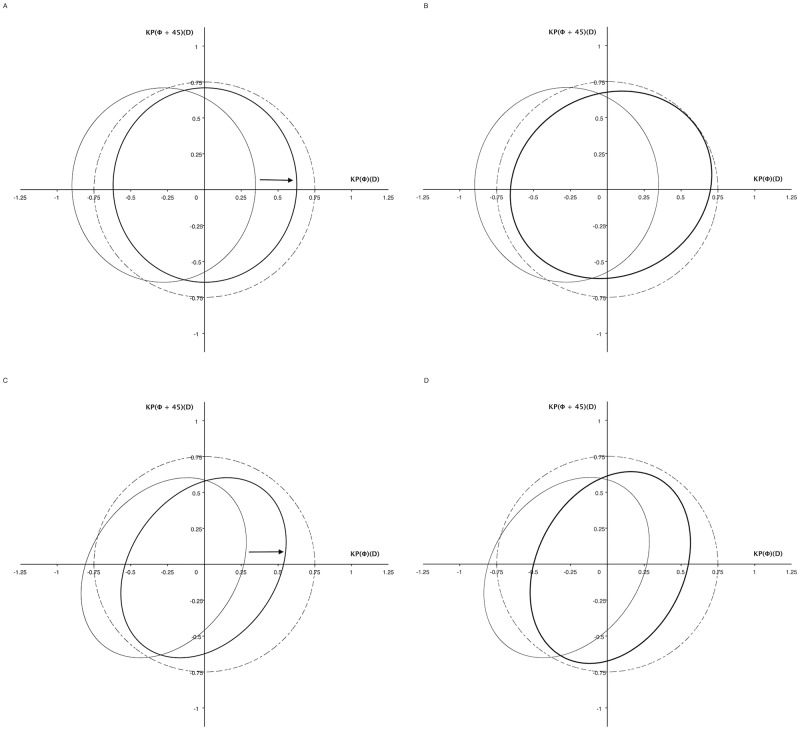
Preoperative (A, B) and postoperative (C, D) adjustment effectiveness shown as pairs of polar values (diopters). By arithmetic adjustment, thin line ellipse moves horizontally (arrows) and reaches thick line ellipse (A, C). By coefficient adjustment, the shape of ellipse is changed (B, D). Dashed thin circle, target circle for 0.75-D magnitude of astigmatism; thin line ellipse, 95% normal region of (TCRP4astig − Kastig); thick line ellipse, 95% normal region of (TCRP4astig − adjusted Kastig); Φ, steep meridian of Kastig in degrees; TCRP4astig, 4-mm-apex zone total corneal refractive astigmatism; Kastig, keratometric astigmatism.

The effectiveness of adjustment was evaluated graphically. Before adjustments, 95% normal regions of (TCRP4astig − Kastig) were not located within the target circle of 0.75-D astigmatic magnitude before and after surgery, indicating that the astigmatic magnitude of (TCRP4astig − Kastig) would exceed 0.75 D in some cases (Fig 4). After arithmetic or coefficient adjustment, the 95% normal region was successfully moved into the target circle preoperatively and postoperatively, indicating that the astigmatic magnitude of (TCRP4astig − adjusted Kastig) would be less than 0.75 D in 95% of cases (Fig 4).

In addition, the total preoperative standard deviations (SDs) were 0.376 for (TCRP4astig − Kastig), 0.376 for (TCRP4astig − arithmetic-adjusted Kastig), and 0.386 for (TCRP4astig − coefficient-adjusted Kastig). The total SD was unaffected by coefficient adjustment (*P* = 0.853, F-test). The total postoperative SDs were 0.344 for (TCRP4astig − Kastig), 0.344 for (TCRP4astig − arithmetic-adjusted Kastig), and 0.352 for (TCRP4astig − coefficient-adjusted Kastig). Again, the total SD was not changed by coefficient adjustment (*P* = 0.875, F-test).

## Discussion

Subjects were included or excluded from the study using SimK and SimKastig of the Pentacam. SimK is the simulated keratometer reading on a ring 15° around the corneal apex, which is approximately equivalent to the 3-mm ring of the anterior surface and agrees with the keratometric power.[7,18] Therefore, discordance between them may indicate measurement errors in the Pentacam or the keratometer. In addition, SimKastig − Kastig showed a strong correlation with TCRP4astig − Kastig; agreement between SimKastig and Kastig was important to remove measurement errors in analyzing the difference between TCRP4astig and Kastig. Thus, inclusion or exclusion by SimK and SimKastig can compensate for test-to-test variability problems of the Pentacam.[25]

To investigate the causes of the differences between TCRP4astig and Kastig, RP4astig and Bastig of the Pentacam and KBastig of the keratometer were introduced. RP4astig is calculated from the front refractive power in the 4-mm zone and reflects the front element of TCRP4astig.[19] Bastig does not perfectly match the back element of TCPR4astig because Bastig is the back keratometric, not refractive, measurement on a ring of 15°, not 4 mm, around the corneal apex.[7,8] Although TCRP4astig and Bastig are calculated using different methods, ray tracing and the paraxial formula, respectively, such differences do not have much effect in normal eyes.[26] However, paraxial formula would be invalid compared with ray tracing if the posterior surface profile of the cornea were altered by refractive surgery.[26] As keratorefractive surgery is known to change only the anterior surface and the posterior surface was confirmed not to be affected by surgery in this study, Bastig may be utilized for the back element of TCRP4astig after surgery.[15,16] Finally, KBastig is an approximation of the back element of Kastig. The meridian of KBastig is the same as that of Kastig and the magnitude of KBastig is proportional to Kastig (Eq 1).

TCRP4astig was significantly different from Kastig before and after surgery (Tables 4 and 5). The difference was in the curvital power, but not in the torsional power, indicating that the difference was due to the astigmatic magnitude, and not the meridian (Table 5). Preoperatively, the back element of Kastig, KBastig, was the only factor to make a difference from TCRP4astig (Table 5). In contrast, both front and back elements of TCRP4astig contributed to the discrepancy of postoperative Kastig (Table 5). As postoperative Kastig was statistically the same as the front refractive astigmatism for the less than 4-mm zone, partially uncorrected astigmatism in the transition zone seemed to affect RP4astig and TCRP4astig (Fig 1).

Previously, the magnitude of Kastig, not the meridian, was adjusted using the arithmetic or coefficient method.[10,11] This study provided grounds for both approaches because the difference between TCRP4astig and Kastig was only in the curvital power along the meridian of Kastig (Table 5). Interestingly, both adjustments were still effective for the cornea modified by myopic keratorefractive surgery (Fig 4). Moreover, adjustment factors of the modified cornea were not statistically different from those of the preoperative cornea (Table 6). In the modified cornea, KBastig of the keratometer became greater than Bastig of the Pentacam along the steep meridian of Kastig while Kastig of the keratometer became less than RP4astig of the Pentacam (Table 5). Increased front refractive astigmatism of the Pentacam counteracted exacerbation of error in keratometric back astigmatism. The elevation of front refractive astigmatism might be more in smaller optical zone diameters (Fig 1). However, the optical zone diameter in the range of 6.3–6.8 mm did not affect the adjustment factor. In addition, the adjustment factors were not statistically different between PRK and LASIK (Table 6 and Fig 3).

The arithmetic adjustment factor of this study, 0.3 D, was smaller than the previous value, 0.5 − 0.6 D, for WTR astigmatism (Table 6).[10] In contrast, the coefficient in this study was not markedly different from the previous coefficient for WTR astigmatism, 0.75, because the 95% confidence interval of the coefficient included 0.75 (Table 6).[11] Previously, the Galilei total corneal power value, which accounts for both anterior and posterior corneal astigmatism, may still underestimate the posterior astigmatism in WTR eyes.[10] The 0.4-D mean posterior astigmatism in this study was unlikely to have been underestimated in comparison of previously reported values, 0.33 D and 0.37 D (Table 1).[7,12] Therefore, further studies are required to determine the accuracy of the arithmetic adjustment factor.

The previous arithmetic and coefficient adjustments are conflicting for high astigmatism in the unmodified cornea. The coefficient adjustment is recommended only in eyes that will receive IOLs with cylinders of 2 D or less and greater IOL cylinder powers are accurately calculated using unadjusted values.[11] However, the arithmetic adjustment is still applied by −0.70 D to −1.00 D for more than 2.00-D of IOL cylinder in WTR astigmatism using Baylor nomograms.[10] In this study, the arithmetic adjustment factor was not changed according to Kastig_magnitude_ within 3.00 D, indicating that the adjustment effect became weaker in high astigmatism, similar to the previous coefficient adjustment (Fig 3A). However, KP(Φ) of (Bastig − KBastig) decreased according to Kastig_magnitude_; this trend may require greater adjustment for higher astigmatism (Fig 2A). Unfortunately, this study could not be applied for Kastig_magnitude_ of more than 3.00 D.

Graphically, arithmetic adjustment involved horizontal movement of the 95% normal region with the same total SD (Fig 4A and 4C). Coefficient adjustment resulted in horizontal movement of the 95% normal region and changed the total SD without statistical significance (Fig 4B and 4D). Consequently, both adjustments did not decrease the total SD or the size of the 95% normal region.

Eventually, direct use of TCRP4astig may provide a simple and effective method in comparison of Kastig adjustment. By addition of 0.7 D, TCRP4 can be converted into the equivalent K reading according to the TCRP4 method and toric IOL power would be calculated directly from 0.7-D added to both K1 and K2 of TCRP4.[18] However, the difference between SimK and the K reading should not be more than 0.5 D, and the discrepancy between SimKastig and Kastig should not be more than 0.5 D in magnitude and 10° in meridian.[18]

This study was limited to WTR astigmatism less than 3.00 D preoperatively and 2.00 D postoperatively. The included subjects may not represent elderly patients with typical senile cataract.

In conclusion, Kastig was different from TCRP4astig in magnitude; both arithmetic and coefficient adjustments of Kastig_magnitude_ were effective in the unmodified and modified cornea. However, neither adjustment decreased the variance in astigmatic disparity. Instead of adjustment, TCRP4astig could be used directly for astigmatic correction in which accordance between SimKastig and Kastig is important. Toric IOL power calculation after keratorefractive surgery would be feasible through the TCRP4 method and TCPR4astig.
